# Stomatal economy: EPF1 balances stomatal density and efficiency

**DOI:** 10.1093/plphys/kiaf440

**Published:** 2025-09-26

**Authors:** Bo Xu

**Affiliations:** Assistant Features Editor, Plant Physiology, American Society of Plant Biologists; ARC Centre of Excellence in Plants for Space and School of Agriculture, Food and Wine, Waite Research Institute, University of Adelaide, Glen Osmond, SA 5064, Australia

Stomata are microscopic pores predominantly distributed on leaf surfaces and act as gatekeepers of plant gas exchange—a process that takes up carbon dioxide (CO_2_) from the atmosphere for photosynthesis and releases oxygen and water vapor into the environment. Each stoma is surrounded by a pair of guard cells. Ion fluxes across their membranes modulate guard cell turgor pressure and volume. This modulation controls the stomatal pore size, thereby regulating stomatal opening and closure, which in turn influences carbon fixation and transpiration rates (Lawson and Matthews 2020).

Manipulating stomatal behavior serves as an effective strategy to optimize water use efficiency (WUE), defined as a ratio of carbon gained through photosynthesis to water loss from transpiration (Nguyen et al. 2023). For instance, guard cell–specific expression of a synthetic blue light–induced potassium (K^+^) channel 1 (BLINK1) in *Arabidopsis thaliana* accelerated stomatal opening and closing speeds by up to 40% in response to light and dark transitions. This faster stomatal responsiveness increased WUE and plant biomass production under fluctuating light (Papanatsiou et al. 2019)—a condition that stimulates natural sun–shade transitions typically observed in the field (Long et al. 2022; Nguyen et al. 2023).

Alternatively, reducing stomatal density through overexpression of epidermal patterning factors (EPFs) has been shown to improve WUE in multiple plant species by inhibiting formation of stomatal lineage cells (Nguyen et al. 2023).

In wheat (*Triticum aestivum*), overexpressing *TaEPF1* reduces leaf stomatal density and enhances WUE (Dunn et al. 2019). However, it is unclear whether *TaEPF1* overexpression affects stomatal development differentially on adaxial (upper) and abaxial (lower) leaf surfaces, since evidence from the Arabidopsis *epf1/epf2* double mutant indicates that EPF-mediated stomatal development is surface specific (Jalakas et al. 2024). Whether such surface-specific alterations also influence whole plant stomatal dynamics remains unknown.

In a recent study published in *Plant Physiology*, Fan et al. (2025) reported that overexpressing *TaEPF1* reduced stomatal formation on both leaf surfaces, with a more pronounced reduction on the abaxial side. Under steady-state red light (1,000 μmol·m^−2^·s^−1^), stomatal conductance and CO_2_ assimilation were reduced in 2 independent *TaEPF1* overexpression lines (1OE3 and 1OE4), consistent with lower stomatal density. Similar to a report by Dunn et al. (2019), the reduction in stomatal density was associated with enhanced WUE in these 1OE lines as compared with wild type plants (Fan et al. 2025). Intriguingly, substituting 10% of red light with blue light (900 + 100 μmol·m^−2^·s^−1^ red + blue) further increased the stomatal conductance, due to stronger stimulation by blue spectral on the stomatal opening (Lawson and Matthews 2020; Fan et al. 2025). Despite this increase in stomatal conductance, the CO_2_ assimilation was only marginally affected, and the WUE was retained at elevated levels in the 1OE lines (Fan et al. 2025).

To further understand the impact of surface-specific stomatal patterning, this study assessed the responsiveness of individual stomata. Stomatal responsiveness was evaluated by calculating stomatal efficiency, defined as the change in normalized stomatal conductance per unit of stomatal density (Δ*g_sw_/*SD) under red light and red–blue light conditions. Under red light, both 1OE lines displayed lower stomatal efficiency than wild type plants, with 16% to 22% reduction on the abaxial surface. However, by substituting 10% of red light with blue light, stomatal efficiency improved dramatically, especially on the abaxial side, where the efficiency increased by approximately 200% on average (Fan et al. 2025). These findings suggest that the lower stomatal density by *TaEPF1* overexpression is compensated by enhancing blue light responsiveness of individual stomata, particularly on the abaxial surface. Collectively, these data highlight a functional trade-off between stomatal density and performance, where fewer stomata are offset by greater per-stoma efficiency (Fan et al. 2025).

This study also raises questions about the basis of surface-specific stomatal efficiency. Fan et al. (2025) proposed that the observed asymmetry in stomatal efficiency—particularly the prominent enhanced stomatal efficiency on the abaxial surface—may be associated with variations in blue light photoreceptor abundance between the surfaces. Moreover, they suggested that a greater intracellular CO_2_ gradient across the leaf, resulting from the substantially reduced abaxial stomatal density, might enhance mesophyll–to–guard cell signaling and promote blue light–induced stomatal opening. These effects highlight an integrated and compensatory mechanism involving CO_2_- and light-dependent signaling pathways, triggered by TaEPF1-mediated stomatal development.

In addition to these speculations, a recent discovery in Arabidopsis may offer a complementary mechanistic insight. Wei et al. (2025) reported that differential expression of 2 inward-rectifying K⁺ channels between leaf surfaces influences stomatal kinetics under ambient light conditions. The voltage-gated inward-rectifying K^+^ channel (KAT1) and the inward-rectifying K^+^ channel (AKT1) are 2 major regulators of K^+^ influx into guard cells, promoting light-induced stomatal opening (Lawson and Matthews 2020; Nguyen et al. 2023). Both genes are expressed in Arabidopsis guard cells; however, *KAT1* is preferentially expressed on the abaxial surface, while *AKT1* is more abundant on the adaxial surface. Computational modeling with the OnGuard platform (Blatt et al. 2022) and genetic complementation suggest that KAT1 contributes more effectively to stomatal opening than AKT1, potentially explaining faster abaxial stomatal responses observed in Arabidopsis (Wei et al. 2025). Whether a similar mechanism underlies the enhanced abaxial stomatal efficiency in *TaEPF1*-overexpressing wheat remains unknown. Given the anatomic and developmental differences between monocots (e.g., wheat) and dicots (e.g., Arabidopsis), it is possible that wheat employs distinct regulatory pathways. Further research is required to determine whether the EFP1-mediated surface-specific stomatal formation influences the expression or function of K^+^ channels in wheat guard cells, thereby modifying the stomatal efficiency.

## Data Availability

No new data were generated or analyzed in support of this research.
